# Physiological Integration Affects Expansion of an Amphibious Clonal Plant from Terrestrial to Cu-Polluted Aquatic Environments

**DOI:** 10.1038/srep43931

**Published:** 2017-03-08

**Authors:** Liang Xu, Zhen-Feng Zhou

**Affiliations:** 1 School of Resource and Environment, Qingdao Agricultural University, Qingdao, 266109, P. R. China; 2 Center for Rural Environmental Studies, Qingdao Agricultural University, Qingdao, 266109, P. R. China

## Abstract

The effects of physiological integration on clonal plants growing in aquatic and terrestrial habitats have been extensively studied, but little is known about the role in the extension of amphibious clonal plants in the heterogeneous aquatic-terrestrial ecotones, especially when the water environments are polluted by heavy metals. Ramets of the amphibious clonal herb *Alternanthera philoxeroides* were rooted in unpolluted soil and polluted water at three concentrations of Cu. The extension of populations from unpolluted terrestrial to polluted aqueous environments mainly relied on stem elongation rather than production of new ramets. The absorbed Cu in the ramets growing in polluted water could be spread horizontally to other ramets in unpolluted soil via physiological integration and redistributed in different organs. The performances of ramets in both terrestrial and aquatic habitats were negatively correlated with Cu intensities in different organs of plants. It is concluded that physiological integration might lessen the fitness of connected ramets in heterogeneously polluted environments. The mechanical strength of the stems decreased with increasing Cu levels, especially in polluted water. We suggest that, except for direct toxicity to growth and expansion, heavy metal pollution might also increase the mechanical risk in breaking failure of plants.

Many exotic invasive plants are also amphiphytes, which span a broad habitat niche ranging from terrestrial to aquatic habitats or *vice versa*^1^,^2^. Furthermore, certain invasive amphiphytes exhibit clonal growth characteristics, such as physiological integration^3^,^4^,^5^. Physiological connections between ramets allow the exchange of resources or signals within the clones^6^,^7^,^8^. It has been repeatedly reported that physiological integration can facilitate the performances of the ramets in either terrestrial or aquatic habitats, especially at the early growth and establishment stages of the juvenile ramets^9^,^10^,^11^. However, only a few studies have explicitly examined how amphibious clonal plants extend populations in aquatic-terrestrial ecotones and take advantage of patchy environments via physiological integration^12^,^13^.

With the changes in environmental stress or resource availability in heterogeneous habitats, physiological integration might become a double-edged sword, which is sometimes beneficial for the recipient ramets at the expense of the donor ramets^12^,^14^,^15^. The benefits and costs of the physiological integration vary between the strength of environmental stress and plant species^16^,^17^. With the fluctuation of environmental factors (e.g., water conditions, resource availabilities, pollutants) in heterogeneous habitats, physiological integration may strengthen the net benefits or *vice versa*^17^. Under severe or long-term environmental stress, the donor ramets are likely to lose fitness to a great extent and terminate supports to the connected receptors^14^. Moreover, the transmission of diseases or spread of toxic substances, (e.g., heavy metals) through interconnected runners might impose an additional cost to the whole clones^9^,^11^,^16^,^18^.

As a widespread problem, the growing level of heavy metal pollution in natural aquatic environments mainly comes from the discharge of municipal sewages and industrial effluent^19^. Excessive intake of heavy metals may lead to direct inhibition of vegetation growth and potential threats to public health throughout the food chain^20^,^21^,^22^. Biotoxicity and bioaccumulation effects of heavy metals on individual plants have been well documented, but such responses of amphibious clonal plants at the population level and interrelated expansion in heterogeneously polluted habitats have rarely been reported^9^,^23^. Within the clones, the clonal growth direction, morphological plasticity and occurrence of physiological integration of one ramet are highly dependent on the other interconnected ramets and associated surroundings^24^. Heterogeneous pollution caused by heavy metals in different types or concentrations presents a challenge to the amphibious clonal plants in aquatic-terrestrial habitats as a result of the translocation of pollutants and the extra costs to donor ramets. Thus, heavy metal pollution in aquatic environments is hypothesized to hinder the expansion of amphibious clonal plants from terrestrial to aquatic habits and subsequent invasion into natural waters.

Amphibious clonal plants usually have a wide ecological amplitude and strong tolerance to heterogeneity with the aid of phenotypic plasticity and locally adapted ecotypes^25^. The opposite of wide distribution and high invasiveness is the low level of genetic variation of certain amphibious clonal plants outside of the primitive environments^26^,^27^,^28^,^29^. As a consequence, the natural selection might be less effective, and the local adaptation is less likely to happen^27^. Researches on phenotypic plasticity of plants are often with respect to responses in individual traits of leaves, roots and stems^5^,^30^,^31^. The plastic responses in different stem traits, for example, length, thickness, stiffness, anatomical structures, should determine the mechanical stability to a great degree, and maintain the other coordinated functions, such as resource transportation. Variation in plasticity in mechanical traits of stems could be affected by wind^31^, sand burial^31^,^32^, trampling^7^,^33^, shading^34^ and flooding^35^. However, little work has been done on such variation in response to different levels of heavy metal pollution. Besides, the sizes and displacements of anatomical structures in stems, for example, collenchyma tissue, ducts, vascular bundles, which are functionally associated with mechanical support and resource mobilization, vary significantly in aquatic and terrestrial plants^36^. The effects of heavy metals on the root and leaf anatomy were well described, but few studies evaluated such effects on anatomical changes in stems^36^,^37^,^38^. The plants discussed before often come from a wide range of distantly related plant groups or single genus, so it is difficult to tell whether these variations are adaptive in truth.

We studied how risk spreading of Cu among the clones affects expansion of populations in aquatic-terrestrial ecotones and associated effects on mechanical and anatomical traits. Following questions are specifically addressed: (1) Do the heavy metal Cu spread among the physically integrated clones in aquatic-terrestrial ecotones ? (2) To what extent is there variation in the expansion of populations in response to Cu spreading ? (3) Whether Cu spreading affects stem traits and increases mechanical risk of ramets exposed to heterogeneous pollution ? Toward this end, the fragments of an amphibious clonal species, *Alternanthera philoxeroides*, were used to simulate the expansion of plants from terrestrial to aquatic habitats at different Cu pollution intensities.

## Results

### Performance of ramets in different habitats

**Figure 1:**
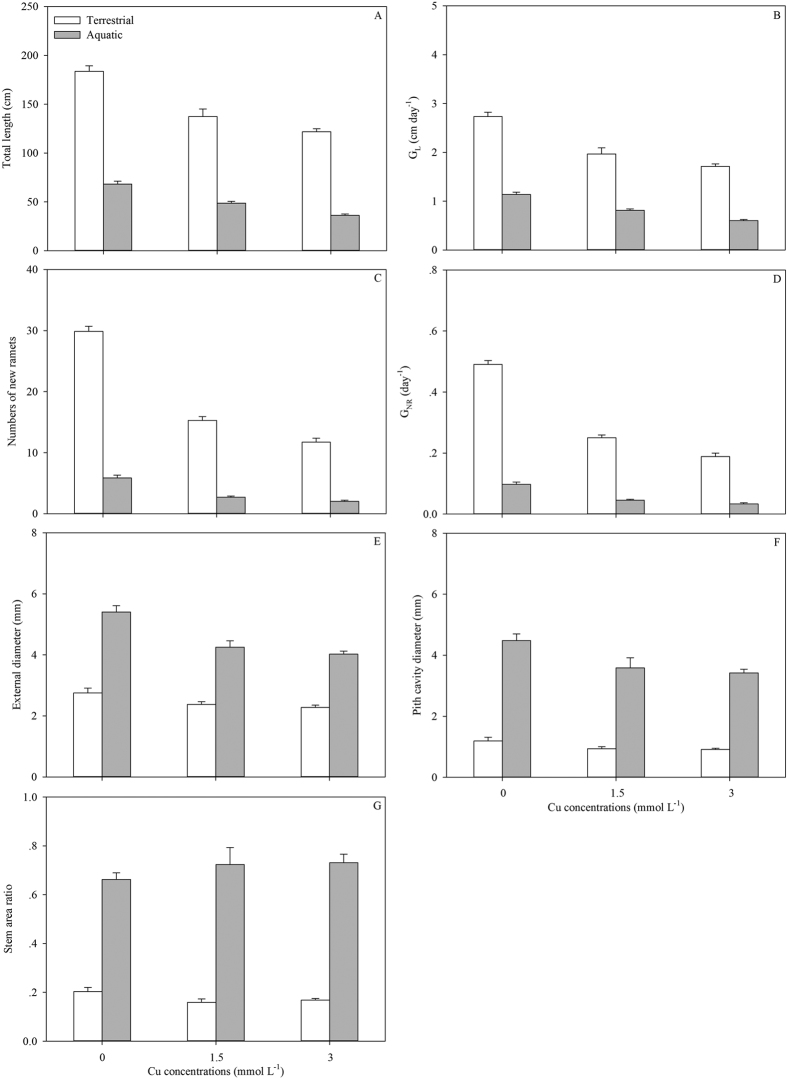
Total stem length (**A**), growth rate of total stem length (G_L_; **B**), numbers of new ramets (**C**), growth rate of new ramets (G_NR_; **D**), external diameter (**E**), pith cavity diameter (**F**) and stem area ratio (**G**) of *A. philoxeroides* grew in terrestrial (white) and aquatic habitats (grey) under different levels of Cu pollution. Data are mean ± SE.

**Figure 2:**
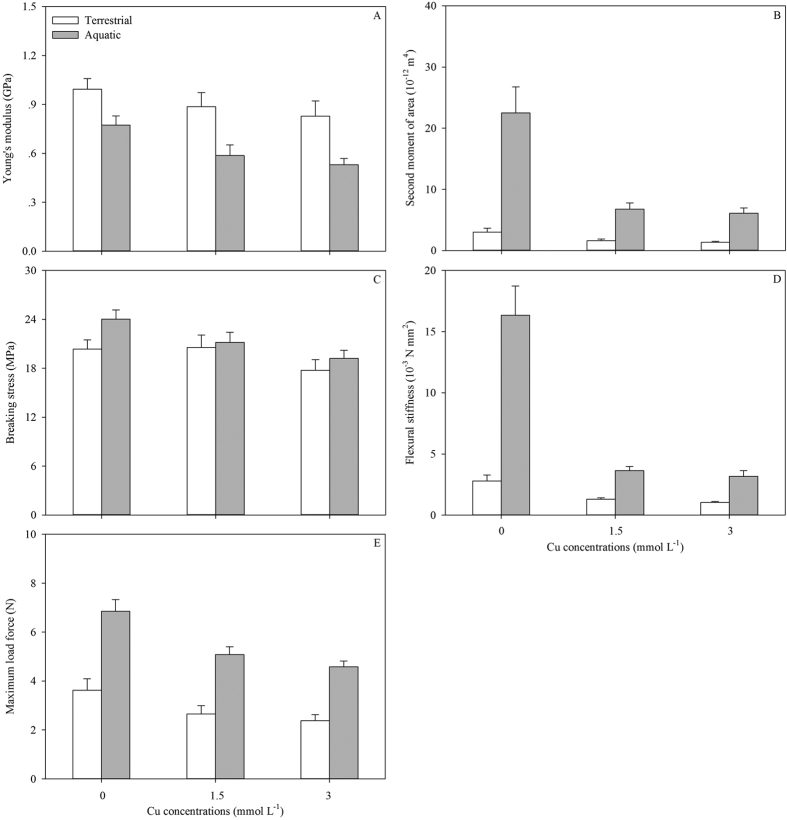
Young’s modulus (**A**), second moment of area (**B**), breaking stress (**C**), flexural stiffness (**D**), maximum load force (**E**) of *A. philoxeroides* grew in terrestrial (white) and aquatic habitats (grey) under different levels of Cu pollution. Data are mean ± SE.

**Figure 3:**
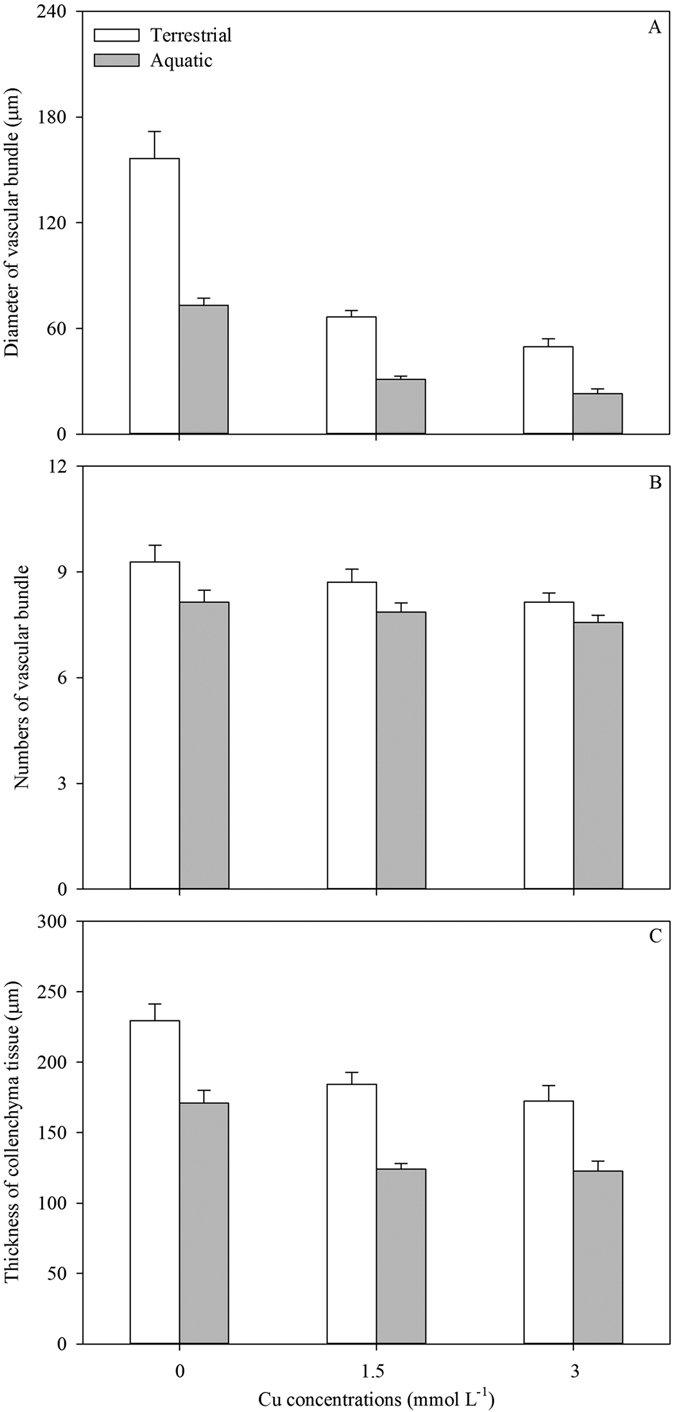
Average diameter of vascular bundles (**A**), numbers of vascular bundles (**B**) and thickness of collenchyma tissue (**C**) of stems of *A. philoxeroides* grew in terrestrial (white) and aquatic habitats (grey) under different levels of Cu pollution. Data are mean ± SE.

**Figure 4:**
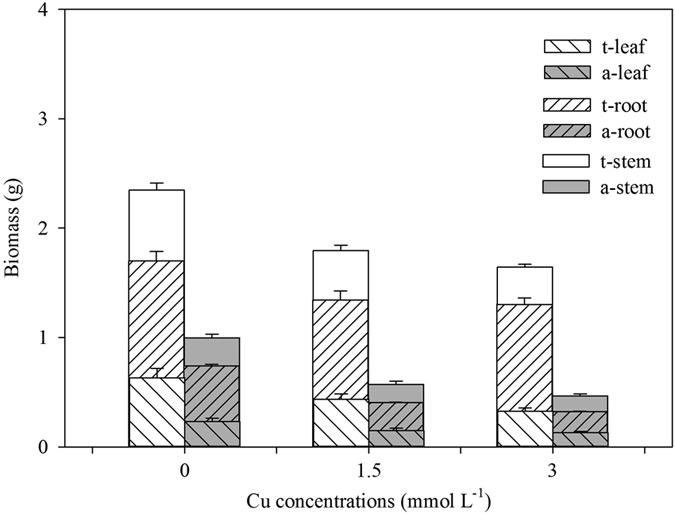
Leaf (right slash), root (left slash) and stem (blank) mass of *A. philoxeroides* grew in terrestrial (white) and aquatic habitats (grey) under different levels of Cu pollution. Data are means ± SE.

Ramets growing in soil exhibited faster growth rates of total stem length (G_L_) and more productions of new ramets (G_NR_) than those in water (Table 1; Fig. 1A,B,C,D), consequently contributing to the longer total stem and more intensive distribution in soil (Table 1; Fig. 1B,D). Comparing the selective internode sections of the same developmental age, both the external diameters (ED) and pith cavity diameters (PCD) of stolons in soil were thinner than those in water (Table 1; Fig. 1E,F). The SAR of the stolons in water was approximately three times as large as that in soil (Table 1; Fig. 1G). Although the stolons in water showed lower tissue stiffness (Young’s modulus, *E*; Table 1; Fig. 2A), the larger second moment of area (*I*) resulted in larger flexural stiffness (*EI*) and maximum load force of the stolons (i.e., the resistance to current rush; Table 1; Fig. 2B,D,E). The stolons in water had less amounts of vascular bundles, smaller diameter of vascular bundles and thickness of collenchyma tissues (Table 1; Fig. 3). The total biomass of ramets in water was smaller than in soil (Table 1; Fig. 4). Finally, the ramets in water allocated more biomass to stems at the cost of roots, while the ramets in soil performed oppositely (Table 1; Fig. 4).

### Cu accumulation and associated effects

**Figure 5:**
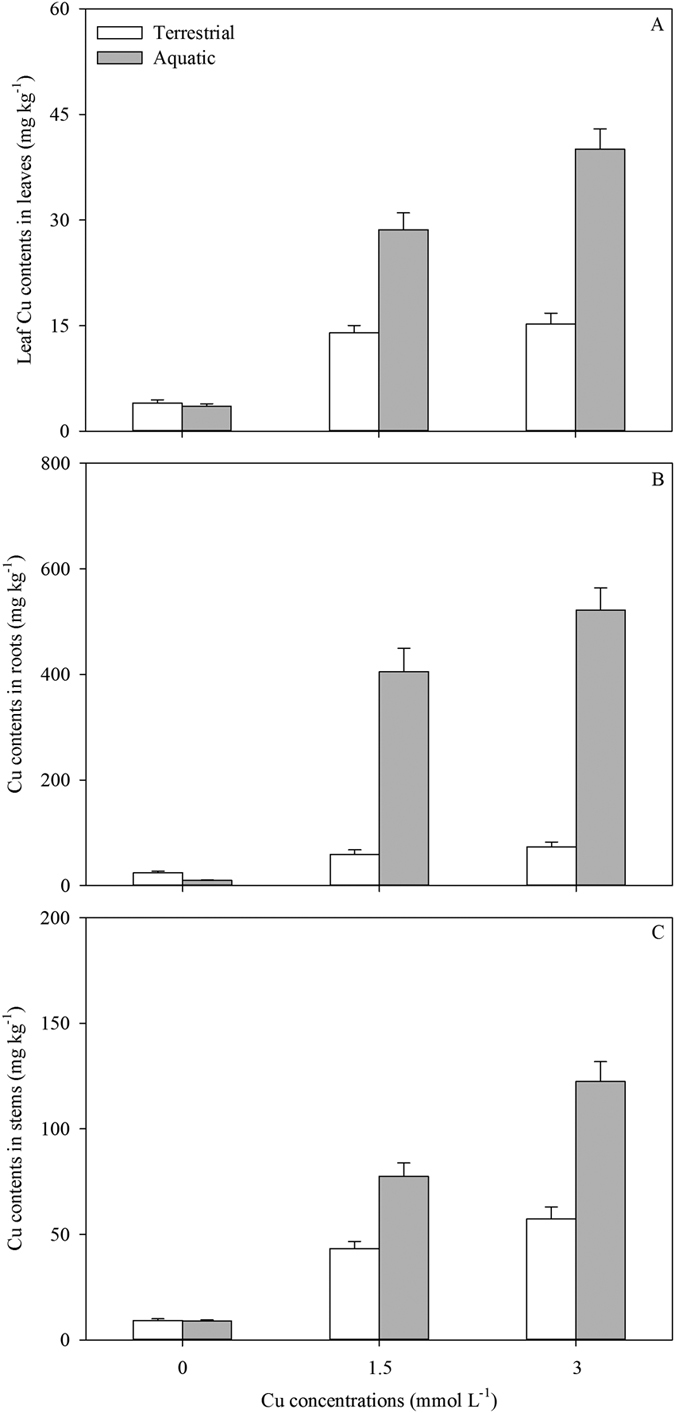
Cu contents accumulated in leaves (**A**), roots (**B**) and stems (**C**) of *A. philoxeroides* grew in terrestrial (white) and aquatic habitats (grey) under different levels of Cu pollution. Data are mean ± SE.

In aquatic habitats, the Cu concentrations in the leaves, roots and stems increased significantly with increasing the level of Cu pollution (Tables 1 and 2; Fig. 5). As a result of Cu spreading via horizontal stolons, the values implied that the Cu concentrations in different tissues of the ramets in soil were significantly affected by Cu levels in water. In terrestrial habitats, connected ramets under pollution had higher Cu concentrations in all the organs than the control plants(Tables 1 and 2; Fig. 5). In both water and soil, the highest Cu concentration was found in the roots (Tables 1 and 2; Fig. 5C). Only a fraction of Cu (*c.*7% in soil and *c.*10% in water) was accumulated in the leaves (Fig. 5A). About 71% Cu was precipitated in the stems rooting in water during the transport, and about 72% Cu was reserved in the perennial roots in soil (Fig. 5B,C).

In the water, only the amount of vascular bundles, SAR, biomass allocation to leaves and stems of the ramets were not significantly affected by the level of Cu pollution (Table 2). Variations in Cu intensities were not only negatively correlated with variations in almost all the traits of the plants in aquatic environment, but also negatively affected the performances of connected ramets in terrestrial habitats (Tables 1 and 2; Figs 1, 2, 3, 4). Except of the amount of VB, PCD, SAR, biomass allocation to leaves, *E, σ*_b_ and *F*, the other traits of the ramets in soil showed significant differences between the three pollution intensities (Table 2).

Meanwhile, significant interactive effects of Cu pollution and different habitats were found in biomass allocation, growth and mechanical traits of plants but not the variations in anatomical structures and morphology (Table 1; Figs 1, 2, 3, 4, 5). Less biomass was invested to the roots in the polluted water, which might reduce uptake of the toxic substances (Tables 1 and 2; Fig. 4). By contrast, more biomass was allocated to the perennial roots in the soil with increasing Cu intensities (Tables 1 and 2; Fig. 4). Though Cu pollution led to decrease in both *E* and *I*, the stem diameter and associated *I* seemed more sensitive to the toxic effect. Cu pollution caused a maximum decrease in *E, I and EI* by *c.* 17%, 56% and 63%, respectively, in terrestrial environments, while the maximum decrease were *c.* 31%, 73% and 81% in aquatic environments (Table 1; Fig. 2A,B,D). The relationships between different traits were shown in Supplementary Table 1 and Supplementary Table 2.

## Discussion

The alien invasive plants generally take an advantage of phenotypic plasticity and genetic differentiation to adapt to heterogeneous habitats. In most cases, it is hard to tell whether variations of plants are plastic responses to heterogeneous environments or genetically based. As a result of low genetic variation of *A. philoxeroides* across China^2^,^26^,^28^,^29^, phenotypic plasticity is likely to play a more important role in the accommodation and invasion in heterogeneous environments. There is a large ecological span from the terrestrial to the aquatic environments. In that case, the organ structures of the amphibious plants should possess both terrestrial and aquatic traits to accommodate the heterogeneity^1^,^12^,^13^. Furthermore, the different traits should be interchanged between different water conditions. It was found in this study that the phenotypic plasticity of *A. philoxeroides* exhibited the adaptability to different habitats in the expansion of populations.

For clonal plants, the well-established ramets can support the growth of physiological connected apical sections and facilitate the broader occupation of space in heterogeneous habitats via physiological integration^1^,^12^,^13^. Elongation of stolons and sprouting of new ramets from the perennial roots or stems are two strategies for *A. philoxeroides* to extend populations in the heterogeneous habitats. The former approach is common but the latter is selective in different environments. Although in the same clones, the ramets in the soil showed faster growth rates of both total stem length and production of new ramets than those in the water. The terrestrial habitats and associated resource distribution are often more heterogeneous than the aquatic habitats. It is also known that the branching intensity commonly increases with the rising levels of resources^39^. For *A. philoxeroides*, the long stem length and strong branching intensities in the terrestrial habitats is beneficial for resource foraging, which lay the foundation for the subsequent invasion and colonization in aquatic environments. In aquatic habitats, the ramets spreading from soil to water mainly depended on the stolon elongation rather than the production of new ramets to extend the populations. Combined with the large areas of pith cavity, the elongated stolons in the water directly enlarge the contact areas with water surface and associated buoyancy. On the other hand, the less biomass allocation to the new ramets, especially the erect shoots, might reduce potential stress to the floatability and risk of submergence caused by excessive biomass.

The large inner cavity of the hollow stems was of great use in maintaining floatability in water, and it would benefit for ventilation across the stems. The conduction efficiency and mechanical safety of the anatomical structures are two important indexes to assess the adaptability of the plants^40^. The thicker conducts usually exhibit a higher efficiency of transportation and a weaker support, whereas the thinner conducts have a lower efficiency, a stronger support and more numbers^36^,^40^. Though the stems in the aquatic environments had thinner thickness of collenchyma tissues, less numbers and smaller diameters of vascular bundles, and related lower stem stiffness (*E*), the much larger *I* resulted in a similarly larger *EI* of the stem sections, which assisted plants to withstand more frequent mechanical impact exerted by waves or currents.

Different plant species absorb heavy metals in the terrestrial and aquatic environments to varied levels and accumulate in different organs^41^. It is reported that the clonal grass *Vallisneria natans* under patchily pollution randomly placed offspring ramets in both polluted and unpolluted patches without selection and avoidance, which made similar population density in different patches^9^. In general, the pollutants in water are distributed more homogeneous than in the terrestrial habitats. It is hard for clonal plants to escape from heavy metal stress and select favorable conditions, especially in the polluted water environments. The existence of physiologically integrated stolons might not only guarantee the resource support from the ramets living in the soil to the ramets rooting in the water, but also potentially allow the risk sharing of toxic stress among the clones^9^,^12^. In the amphibious clonal plant *A. philoxeroides*, the pollutant Cu can spread among the clones rooting in different habitats. In the contaminated aquatic environments, it is supposed that the assimilated Cu firstly moved upward from the adventitious roots to the leaves and the stems. In addition, a portion of Cu was transported via horizontal stolons to the ramets in uncontaminated terrestrial habitats. Further, the Cu was distributed basipetally to belowground perennial roots, and acropetally to the leaves and the stems. Thus, the Cu and associated stress were spread in the whole clones.

For most plant species, the heavy metal accumulations were found to be higher in the roots located in the polluted habitats than in the shoots^18^. In accordance with intraclonal division of labor in the clones, the pollutants might lead to less biomass investment in roots in the polluted environments to reduce absorption of pollutants, while plants in the unpolluted environments might allocate more biomass to roots to draw plenty of nutrients which could be shared among the whole clones^9^. In this study, the adventitious roots in the polluted water, which was high in Cu level comparing with that of the unpolluted soil, primarily accumulated high concentrations of Cu. The perennial roots in the soil are the core organs in resource storage^2^,^26^. It is supposed that the pollutant Cu should be stored in the perennial roots like resources, resulting in a high accumulation in the ramets in soil. Besides, no more than *c.* 10% Cu was transported to leaves, and up to *c.* 72% Cu was deposited in the stems in polluted water. Indeed, the deposition of non-degradable heavy metals in the non-photosynthetic sections of the plants should be an important strategy to tolerate the toxicity^37^. The accumulations of Cu in different tissues might become toxic when the concentrations exceed certain intensities^42^. Whether in direct contact or translocation, the growth and extension of the ramets in both the terrestrial and aquatic habitats were severely inhibited under low- and high-levels of Cu pollution. The spreading and accumulation of Cu between all the interconnected ramets might increase the stress to the ramets without contact with the pollutant. In view of the translocation of resources (e.g. photosynthates), the support to the ramets under direct pollution might decrease the fitness of the donor ramets^12^,^14^,^15^.

Heavy metals accumulated in the tissues can affect the balance of the hormones, and subsequently influence the tissue development and morphogenesis^37^. Changes in anatomical structures should be functionally coordinated with the hydraulic capacity and resources translocation. The Cu ions were exported through the transpiration stream in the vascular bundle. The accumulation speed of heavy metal ions in the anatomical structures was faster than that of removal, which develops osmotic stress favoring the flows in opposite direction in the vascular bundles^38^. Conditional on the plasticity in anatomy, *A. philoxeroides* exposed to pollution could develop the modified tissues which allowed greater adaptability to the stress. Reduction in the sizes and amounts of conducting elements is considered as an adaptive strategy to secure the resource flow^37^.

The anatomical structure elements, such as sclerenchyma, collenchymas and vascular bundles are correlated with bending rigidity and strength of stems, helping to maintain mechanical stability, or withstand the tensile force exerted by currents^36^. The toxic effects of pollutant Cu on anatomical and related mechanical traits were represented by the negative correlation between the traits of the stems and Cu accumulation. It has been documented that there are linear relationships have been found between the current-induced drags and plant length, and between the drags and flow speed^36^. Those mean that the sections of stems far away from the basal sections were likely to take greater mechanical risk in tensile failure caused by currents or waves. Ecosystems polluted by heavy metals are characterized to impose important constraints on growth, production and population expansion of plants^9^, which might produce short, sparse and mechanically weak stems. Such stems should be more vulnerable to breaking, especially near the distal end.

We conclude that Cu pollution may induce stress directly to ramets in polluted aquatic environments. The spreading and accumulation of Cu via physiological integration lowered the performance of interconnected ramets in unpolluted terrestrial habitats by direct toxicity or the exportation of resources as the donor^12^,^14^,^15^. It is suggested that the toxicity of Cu might increase the mechanical risk in tensile failure, especially near the distal end of the stems. The population expansion of the plants in both aquatic and terrestrial habitats was negatively correlated with the levels of Cu pollution in the water. The results add to our understanding of considering coordinated traits when analyzing the phenotypic plasticity of plants in heterogeneous environments.

## Material and Methods

### Ethics statement

The plant material, *Alternanthera philoxeroides*, used in this experiment was collected from an uncultivated area along the bank of the Moshui River in Qingdao, Shandong Province, China. The amphibious clonal plant is common and naturally distributed in this area. Thus, specific permission was not required for the collection of this herbaceous plant or to visit the location where we collected the samples. This work in both field and experiment did not involve any endangered or protected species.

### Focal species and study site

*Alternanthera philoxeroides* (Mart.) Griseb (Amaranthaceae) is an amphibious perennial herb originating from Parana River region in South America^1^,^2^,^3^,^26^. As one of the most detrimental invaders in China, it is widely distributed in aquatic (e.g., rivers, lakes, canals) and terrestrial habitats (e.g., crop lands, lawn) and the junctions (e.g., river banks,) in tropic and subtropical area. Its reproduction mainly relies on vegetative propagation with perennial roots in soil and stolons, but not viable seeds^1^,^2^,^3^,^10^. There are perennial roots in terrestrial habitats which store a large amount of nutrients to support growth and population regeneration. In the water, the stolons floating in the water can only produce adventitious roots^2^,^26^. Molecular marker analyses have proved that the genetic variation of *A. philoxeroides* both within and among populations is extremely low across China^2^,^26^,^28^,^29^.

### The experiment and measurements

On 21 June 2014, we collected clonal fragments of *A. philoxeroides* along the banks of Moshui River in Qingdao, Shandong Province, China. These fragments were propagated vegetatively in soil in a greenhouse at Qingdao Agricultural University, China.

On 29 May 2015, a number of small clonal fragments of similar size, each of which consisted of a perennial root, and a stolon with an apex, were severed from the clonal populations and rooted in plastic pots (length 60 cm × width 34 cm × height 18.5 cm, effective volume 29.38 L). Each pot simulating a terrestrial habitat was filled with a 1:1 mixture (composed of soil and sand) and four grams of solid slow-release fertilizer (16N-11P_2_O_5_-11K_2_O-3MgO + trace elements, 3-4 months, Osmocote Exact, Scotts International B.V., Heerlen, the Netherlands). To leave enough space for growth and extension, the primary rooting position from the edge of pot was about 15 cm. After two weeks, the mean total length of the stem reached 19.42 ± 0.49 cm, and the main stolons had grown out of the pot edge. Another pot was filled with tap water to simulate an aquatic habitat. The pots simulating the terrestrial and aquatic environments were placed next to each other. Different doses of Cu was added as the sulfate (CuSO_4_·5H_2_O) into the tap water and mixed thoroughly to simulate the polluted water environments. The three Cu concentrations were 0 for the control, 1.5 mmol L^−1^ for the low-level pollution and 3 mmol L^−1^ for the high-level pollution, respectively. Each treatment was repeated seven times. During the experiment, the water surface elevations in the pots simulating aquatic environments were maintained at a similar height, and the fragments rooted in the soil were watered regularly,

Plants in terrestrial and aquatic habitats were harvested separately on 12 August 2015. Total length of all stems, including the horizontal stolons and the erect stems, and total numbers of all new ramets (NR in short) were measured. Then plants were separated into leaves, roots and stems with sharp scissors. The roots were carefully washed. The traits of the fourth to sixth internodes severed from the main stolons in both terrestrial (counting from the initial rooting position) and aquatic habitats(counting from the edge of the pots) were measured. The external diameters (ED in short) and the pith cavity diameters (PCD in short) were measured three times and the average values were taken. A variety of mechanical traits of the stems (to be exact, represented by the fourth to sixth internodes) were tested and calculated. Young’s modulus (*E*) indicating stiffness of an elastic material 43, breaking stress (*σ*_b_) and maximum load force (*F*) quantifying the resistance of tissue to rupture, of stems were directly measured with a universal electromechanical testing machine (Type 5540; Instron, Norwood, Massachusetts, USA) by a three-point bending technique (for details see ref. 7,34). The distance between supports was adjusted such that it was always approximately 15 times the average diameter of the stem section. The second moment of area (*I*, m^4^) describing the geometric contribution to stiffness of the stem^7^ and flexural stiffness (*EI*, N m^−2^) of the rigidity of a stem cross section were calculated with ED and PCD^43^. After the test of mechanical traits, the rest fresh internodes were made into free-hand sections with sharp razor blades and observed with a dissecting microscope (Olympus BH-2, Tokyo, Japan). The average thickness of collenchyma tissue (hereafter CT in short), amounts and average diameters of vascular bundles (hereafter VB in short) in one section were measured and recorded. After all of the measurements with fresh tissues, the dry biomass of leaves, roots and stems were obtained after drying in a stove at 70 °C for 48 h. The last analysis was the Cu concentrations in different organs. Dry samples of leaves, roots and stems were ground with a ball mill (DECO-PBM-V-4L, Changsha, Hunan Province, China) and dried to a constant weight. The homogenized samples and 25 ml H_2_O_2_/HNO_3_ at a ratio of 1:4 (*v/v*) were put into Teflon crucibles (effective volume 100 ml) together for eight hours. The crucibles were sealed using steel cans and put in a stove at 80 °C for one hour. After the steel cans cooled, the crucibles were taken out and heated on an electrical heating panel (MWJ-3020, Wuxi, Jiangsu Province, China) at 120 °C for two hours. The purpose was to remove excess acid from the solution. Cu concentrations in the extracts were tested using an inductively coupled plasma-optical emission spectrometry (Optima 8000, Perkin Elmer, Massachusetts, the USA).

### Data processing and analysis

The mean absolute growth rates of total stem length (G_L_) and total numbers of new ramets (G_NR_) throughout the whole experiment were calculated. The stem area ratio (SAR in short) was the square of the ratio of PCD to ED.

Two-way ANOVA was used to test for the effects of heterogeneous environments and Cu pollution on the different stem traits and Cu accumulation traits. One-way ANOVA was used to test the variations of the ramets in soil and the ramets in water under different intensities of Cu pollution, separately. A principal component analysis was used to classify the different traits and distinguish the indicative property of plants for Cu^2+^ pollution. SPSS 21 (IBM Inc., Armonk, New York, USA) was used for all of the statistical analyses. *P* < 0.05 was used as the significance level. The regression equations in the figures were generated using Sigmaplot 12.5 (Systat Software Inc., Erkrath, Germany).

## Additional Information

**How to cite this article:** Xu, L. and Zhou, Z.-F. Physiological Integration Affects Expansion of an Amphibious Clonal Plant from Terrestrial to Cu-Polluted Aquatic Environments. *Sci. Rep.* **7**, 43931; doi: 10.1038/srep43931 (2017).

**Publisher's note:** Springer Nature remains neutral with regard to jurisdictional claims in published maps and institutional affiliations.

## Supplementary Material

Supplementary Information

## Figures and Tables

**Table 1:** Results of two-way ANOVA showing the effects of Cu pollution (P; d*f* = 2) and different habitats (H; d*f* = 1) on the anatomical, morphological, biomass allocation, growth, Cu accumulation and mechanical traits of *A. philoxeroides*.

	Anatomical traits			Morphology				
	Amount of VB	Diameter of VB	Thickness of CT	ED	PCD		SAR	
Pollution	3.41*	106.48***	20.40***	18.46***	8.65**		0.08	
Habitat	10.23**	128.33***	58.85***	320.95***	501.82***		424.71***	
P*H	0.38	0.03	0.19	1.04	1.02		1.63	
	**Biomass allocation**			**Growth**				
	**Root ratio**	**Leaf ratio**	**Stem ratio**	**Length**	**G**_**L**_	**NR**	**G**_**NR**_	**Biomass**
Pollution	0.39	0.27	3.78*	88.34***	75.47***	99.84***	232.33***	33.68***
Habitat	996.11***	1.16	1097.62***	1120.23***	655.14***	813.76***	1165.63***	392.81***
P*H	20.39***	3.38*	5.99*	4.68*	0.95	0.55	97.37***	0.74
	**Cu accumulation**			**Mechanical traits**				
	**Leaf Cu**	**Root Cu**	**Stem Cu**	***E***	***σ***_**b**_	***F***	***I***	***EI***
Pollution	242.44***	265.39***	420.76***	5.54**	4.66*	13.21***	22.63***	62.62***
Habitat	46.66***	101.52***	43.99***	24.10***	3.67	79.75***	142.84***	165.82***
P*H	18.47***	95.25***	12.47***	0.57	0.83	1.12	4.28*	6.08**

Meanings of abbreviations of morphology can be found in the Experiment and Measurements section of Methods. *F* values and significance levels are given (***P < 0.001, **P < 0.01, *P < 0.05). Data were natural logarithm transformed before analyses.

**Table 2:** Results of one-way ANOVA showing the effects of Cu pollution on the anatomical, morphological, biomass allocation, growth, Cu accumulation and mechanical traits of the ramets in terrestrial and aquatic habitats, separately.

	Anatomical traits			Morphology				
	Amount of VB	Diameter of VB	Thickness of CT	ED	PCD		SAR	
Terrestrial	2.32	50.27***	8.18**	4.81*	3.30		2.93	
Aquatic	1.09	56.39***	14.91***	16.04***	5.67*		0.64	
	**Biomass allocation**			**Growth**				
	**Root ratio**	**Leaf ratio**	**Stem ratio**	**Length**	**G**_**L**_	**NR**	**G**_**NR**_	**Biomass**
Terrestrial	7.92**	1.80	10.57**	31.07***	32.23***	177.12***	189.29***	8.27**
Aquatic	14.16***	1.87	0.26	58.57***	58.51***	43.19***	43.19***	34.72***
	**Cu accumulation**			**Mechanical traits**				
	**Leaf Cu**	**Root cu**	**Stem Cu**	***E***	***σ***_**b**_	***F***	***I***	***EI***
Terrestrial	56.61***	17.30***	126.04***	1.03	1.37	3.28	4.89*	15.91***
Aquatic	227.02***	459.36***	338.09***	5.51*	4.63*	11.15***	18.09***	51.51***

Meanings of the abbreviations can be found in the Experiment and Measurements section of Materials and Methods. *F* values and significance levels are given (***P < 0.001, **P < 0.01, *P < 0.05). Data were natural logarithm transformed before analyses.
