# Supplementary material for: Physiological Integration Affects Expansion of an Amphibious Clonal Plant from Terrestrial to Cu-Polluted Aquatic Environments
Source: Sci Rep. 2017 Mar 8;7:43931. doi: 10.1038/srep43931 (PMC5341073; doi:10.1038/srep43931)
Supplement: Supplementary Information [file srep43931-s1.pdf]

Supplementary Information

Physiological Integration Affects Expansion of an Amphibious Clonal Plant from Terrestrial to Cu-Polluted Aquatic Environments

Liang Xu[1, 2*], Zhen-Feng Zhou[1, 2]

1 School of Resource and Environment, Qingdao Agricultural University, Qingdao 266109, PR China

2 Center for Rural Environmental Studies, Qingdao Agricultural University, Qingdao 266109,PR China

* Corresponding author. E-mail: xuliang@qau.edu.cn

Tel: +86-532- 86080571

Tables

Supplementary Table 1. Results of principal component analysis showing the explanation for variance and rotated matrix.

Component	Initial eigenvalues			Rotated matrix				
	Total	Percentage of variance (%)	Cumulative of variance (%)	Parameter	Component 1	Component 2	Component 3	Component 4
1	12.065	54.842	54.842	Leaf Cu	-0.814	-0.128	0.413	-0.197
2	4.393	19.970	74.812	Root Cu	-0.767	0.045	0.491	-0.237
3	1.679	7.632	82.444	Stem Cu	-0.811	-0.197	0.370	-0.267
4	1.332	6.054	88.498	Length	0.828	-0.508	-0.145	-0.003
5	0.697	3.167	91.664	G_L	0.855	-0.447	-0.126	0.010
6	0.629	2.859	94.523	NR	0.877	-0.409	0.098	0.064
7	0.288	1.307	95.830	G_{NR}	0.882	-0.398	0.103	0.052
8	0.230	1.046	96.876	ED	-0.240	0.952	0.089	0.003
9	0.152	0.689	97.565	PCD	-0.400	0.869	0.160	0.073
10	0.142	0.647	98.212	SAR	-0.588	0.679	0.288	0.057
11	0.097	0.441	98.653	Amount of VB	0.677	-0.056	0.139	-0.220
12	0.082	0.373	99.026	Diameter of VB	0.847	-0.091	0.071	0.261
13	0.075	0.343	99.369	Thickness of CT	0.892	-0.149	-0.008	-0.091
14	0.053	0.243	99.612	Biomass	0.804	-0.485	-0.179	0.029
15	0.045	0.204	99.815	Leaf Ratio	0.046	0.047	0.839	0.107
16	0.022	0.100	99.915	Root ratio	0.463	-0.724	-0.470	-0.036
17	0.009	0.041	99.956	Stem ratio	-0.511	0.765	0.261	0.007
18	0.007	0.030	99.986	E	0.513	-0.359	-0.083	0.715
19	0.002	0.011	99.997	σ_b	0.007	0.305	0.089	0.887
20	0.000	0.002	99.999	F	-0.182	0.727	0.184	0.562
21	0.000	0.001	100.000	I	0.033	0.905	-0.247	-0.003
22	0.000	0.000	100.000	EI	0.122	0.897	-0.272	0.132

Supplementary Table 2. Results of principal component analysis showing the correlation matrix.

	Leaf Cu	Root Cu	Stem Cu	Length	G_L	NR	G_{NR}	ED	PCD	SAR	Amount of VB	Diameter of VB	Thickness of CT	Biomass	Leaf Ratio	Root ratio	Stem ratio	*E*	σ_b	*F*	*I*	*EI*
Leaf Cu	1.000	.886	.892	-.649	-.669	-.615	-.615	.108	.268	.511	-.419	-.687	-.693	-.658	.227	-.466	.435	-.523	-.179	.032	-.221	-.325
Root Cu	.886	1.000	.861	-.711	-.715	-.616	-.615	.270	.401	.620	-.453	-.603	-.686	-.723	.260	-.625	.596	-.621	-.159	.134	-.089	-.217
Stem Cu	.892	.861	1.000	-.619	-.651	-.605	-.607	.045	.192	.419	-.417	-.705	-.664	-.633	.241	-.372	.330	-.555	-.248	-.086	-.253	-.373
Length	-.649	-.711	-.619	1.000	.995	.935	.931	-.686	-.787	-.852	.554	.750	.769	.978	-.140	.805	-.824	.604	-.158	-.550	-.398	-.318
G_L	-.669	-.715	-.651	.995	1.000	.940	.937	-.631	-.739	-.812	.560	.781	.774	.975	-.136	.756	-.773	.597	-.126	-.494	-.352	-.269
NR	-.615	-.616	-.605	.935	.940	1.000	.999	-.588	-.683	-.741	.534	.865	.814	.887	.058	.644	-.709	.622	-.071	-.400	-.358	-.273
G_{NR}	-.615	-.615	-.607	.931	.937	.999	1.000	-.577	-.675	-.734	.541	.863	.820	.882	.062	.638	-.704	.609	-.075	-.399	-.351	-.266
ED	.108	.270	.045	-.686	-.631	-.588	-.577	1.000	.969	.832	-.211	-.281	-.361	-.658	.100	-.833	.866	-.467	.303	.739	.813	.790
PCD	.268	.401	.192	-.787	-.739	-.683	-.675	.969	1.000	.930	-.293	-.387	-.479	-.761	.116	-.882	.915	-.470	.347	.768	.683	.661
SAR	.511	.620	.419	-.852	-.812	-.741	-.734	.832	.930	1.000	-.377	-.500	-.628	-.837	.133	-.909	.938	-.532	.287	.692	.443	.404
Amount of VB	-.419	-.453	-.417	.554	.560	.534	.541	-.211	-.293	-.377	1.000	.355	.646	.510	.037	.338	-.374	.241	-.055	-.231	-.132	-.091
Diameter of VB	-.687	-.603	-.705	.750	.781	.865	.863	-.281	-.387	-.500	.355	1.000	.717	.710	.022	.385	-.420	.621	.158	-.054	-.070	.022
Thickness of CT	-.693	-.686	-.664	.769	.774	.814	.820	-.361	-.479	-.628	.646	.717	1.000	.722	.003	.558	-.601	.479	-.124	-.301	-.136	-.052
Biomass	-.658	-.723	-.633	.978	.975	.887	.882	-.658	-.761	-.837	.510	.710	.722	1.000	-.126	.787	-.809	.609	-.131	-.525	-.361	-.277
Leaf Ratio	.227	.260	.241	-.140	-.136	.058	.062	.100	.116	.133	.037	.022	.003	-.126	1.000	-.384	.122	.018	.164	.182	-.062	-.071
Root ratio	-.466	-.625	-.372	.805	.756	.644	.638	-.833	-.882	-.909	.338	.385	.558	.787	-.384	1.000	-.963	.515	-.266	-.724	-.514	-.453
Stem ratio	.435	.596	.330	-.824	-.773	-.709	-.704	.866	.915	.938	-.374	-.420	-.601	-.809	.122	-.963	1.000	-.559	.238	.725	.570	.508
E	-.523	-.621	-.555	.604	.597	.622	.609	-.467	-.470	-.532	.241	.621	.479	.609	.018	.515	-.559	1.000	.484	.021	-.276	-.125
σ_b	-.179	-.159	-.248	-.158	-.126	-.071	-.075	.303	.347	.287	-.055	.158	-.124	-.131	.164	-.266	.238	.484	1.000	.710	.248	.362
F	.032	.134	-.086	-.550	-.494	-.400	-.399	.739	.768	.692	-.231	-.054	-.301	-.525	.182	-.724	.725	.021	.710	1.000	.569	.624
I	-.221	-.089	-.253	-.398	-.352	-.358	-.351	.813	.683	.443	-.132	-.070	-.136	-.361	-.062	-.514	.570	-.276	.248	.569	1.000	.975
EI	-.325	-.217	-.373	-.318	-.269	-.273	-.266	.790	.661	.404	-.091	.022	-.052	-.277	-.071	-.453	.508	-.125	.362	.624	.975	1.000
